# Cloning and expression analysis of *GATA*1 gene in *Carassius auratus* red var

**DOI:** 10.1186/s12863-021-00966-3

**Published:** 2021-03-18

**Authors:** Yusu Tian, Yuandong Sun, Mi Ou, Xiaojuan Cui, Dinggang Zhou, Wen’an Che

**Affiliations:** 1 Hunan Key Laboratory of Economic Crops Genetic Improvement and Integrated Utilization, School of Life Sciences, Hunan University of Science and Technology, Xiangtan, 411201 Hunan People’s Republic of China; 2 Key Laboratory of Tropical and Subtropical Fishery Resources Application and Cultivation, Ministry of Agriculture, Pearl River Fisheries Research Institute, Chinese Academy of Fishery Sciences, Guangzhou, 510380 Guangdong, Hunan People’s Republic of China

**Keywords:** *Carassius auratus* red var.,, *GATA*1, Cloning, Methylation, Expression characteristics

## Abstract

**Background:**

*GATA*1 is a key transcription factor in the GATA family, and promotes the differentiation and maturation of red blood cell, which is essential for normal hematopoiesis.

**Results:**

Our results showed that the cDNA sequence of *GATA*1 was 2730 bp long encoding 443 amino acids. qRT-PCR analysis demonstrated that *GATA*1 had the highest expression in testis (T), followed by pituitary (P) and spleen (S). *GATA*1 gene expression in *C. auratus* red var. embryo from the neuroblast stage (N) to the embryo hatching (H) changes continuously; and the gene expression levels of nonylphenol (NP)-treated and those of control embryos were significantly different. Moreover, Methylation levels of *GATA1* gene in NP-treated embryos were higher than those in control embryos, indicating that NP affected *GATA1* methylation.

**Conclusions:**

Our study provides cues for further studying the roles of *GATA*1 gene in fish development, and suggested a potential molecular mechanism by which NP leads to abnormal development of fish embryos.

**Supplementary Information:**

The online version contains supplementary material available at 10.1186/s12863-021-00966-3.

## Background

Nonylphenol (NP), an environmental hormone that mimics estrogen and binds to its receptors in the cell, interferes with endocrine metabolism and has toxic effects on animals [1]. Studies demonstrated that NP was detrimental to reproduction. For example, NP causes male reproductive dysfunction, damages the development of testis, and leads to the decline of male fertility and sperm counts [2]. A low 4-NP (a typical isomer of para-NP) dosage induced uterine nutrition response in prepuberty rats, but not in ovariectomized adult rats [3]. Tanaka et al. showed that *Rivulus marmoratus* had abnormal gonadal development and testis insufficiency when exposed to NP [4]. In *Oryzias latipes*, the percentage of motile spermatozoa after sperm exposure to NP decreased dramatically [5]. However, although 4-NP did affect sperm production in *Oncorhynchus mykiss*, it showed no effect on sperm density, motility and fertility [6]. NP not only affected adult fish, but also interfered with fish embryonic development. When *Puntius conchonius* embryos were exposed to NP, they showed developmental abnormalties such as egg coagulation, spinal deformity, and delayed development [7]. NP had drastic toxicity to development of goldfish embryos, which showed higher sensitivity to low concentrations of NP than adult fish [8]. 4-NP also affected the development of embryos and larvae of *Oncorhynchus mykiss* at the end of the yolk sac stage, reducing their survival rate [6]. In NP-exposured zebrafish embryos, the distribution of PGCs along the anterior–posterior axis in 24-h-old embryos changed, which may influence the juvenile and adult gonadal structures [9].

There have been some studies on the effects of NP on gene expression in vivo. Xia et al. reported that the expression of cy5 and cy3 in the rat was down-regulated under NP exposure [10]. When *Chironomus riparius* larvae were treated with NP, the expression level of CrEcR was significantly up-regulated, through which nonylphenol might have significant implications in various developmental stages of *C. riparius* [11]. P353-NP (a typical isomer of para-NP) caused embryonic dysplasia in zebrafish (*Danio rerio*), with the expression of *ntl* and *spt* unchanged but that of *tbx6* significantly increased [12]. Nonylphenol exposure reduced Na^+^/K^+^-ATPase activity, plasma cortisol and triiodothyronine levels in *Salmo salar* gills [13]. In addition, another study in *Salmo salar* suggests NP could regulate the hepatic enzyme activities that were mediated by Cyp3a and Cyp1a1 through Pxr and Ahr. Furthermore, NP might have impacts on metabolism of both endogenous and exogenous substrates [14]. Paolo et al. found that significantly higher PPARα mRNA levels in *Solea solea* were associated with 4-NP treatment for 3 days while the highest dose of 4-NP in their study also led to up-regulation of retinoid X receptor α (RXRα) transcription [15].

GATA1 is a key transcription factor for erythropoiesis, and it contains three conserved functional domains: C-zinc finger, N-zinc finger, and N-terminal activation [16]. The two zinc finger domains are responsible for DNA binding and protein-protein interactions, which allow them to recognize typical *GATA* binding sites with a consensus sequence WGATAR [17]. *GATA*1 is indispensable in differentiation of erythroid cells and megakaryocytes. In the development of erythroid cells, GATA1 functions early in megakaryocytes. *GATA*1 controls terminal maturation and its deficiency induces proliferation [18]. Galloway established a transcriptional hierarchy dependent on GATA in the process of hematopoiesis, and demonstrated that GATA1 played an integral role in the fate determination of myelo-erythroid lineage during embryogenesis [19]. Chan et al. found that reduced hematopoiesis in *Choonodraco hamatus* was associated with miR-152-mediated down-regulation of *GATA*1 [16]. More importantly, studies have found abnormal localization of P-selectin induced by *GATA*1 (low) mutations, and increased pathological interactions with leucocytes as well, which were responsible for increased thrombosis in mice [20].

*Carassius auratus* red var. fulfills our basic requirements of experimental animals. It is convenient for artificial breeding, easy to discover and eliminate mutant individuals, and highly sensitive to NP [21, 22]. *C. auratus* red var. embryos developed malformations under NP stress, such as spine curvature, tail deformity, pericardial abnormalities and thrombosis [23]. Our previous transcriptome study revealed that *GATA*1 expression in *C. auratus* red var. embryos was affected by NP-treatment, which may be one of the causes for embryonic malformation *C. auratus* [23]. In this study, we cloned and sequenced the full-length *GATA1* cDNA in *C. auratus* red var., and conducted bioinformatics analysis. In addition, we used realtime fluorescence quantitative PCR (qRT-PCR) to explore expression patterns of *GATA*1 in different tissues of *C. auratus* red var. and transcriptional changes of *GATA*1 after exposure to different concentrations of NP. Moreover, we measured differences in DNA methylation levels of the *C. auratus* red var. embryos between the NP treatment groups and the control ones at various developmental stages, and measured NP treatment effects on *GATA*1 methylation. This study investigated the expression of *GATA*1 gene in abnormal development of *C. auratus* red var. embryos under NP stress, and explored the relationship between thrombosis and *GATA*1 gene in malformed embryos. Our study provides cues for further research on the molecular mechanism of embryo development deformity in *C. auratus* red var. caused by NP.

## Results

### Analysis of GATA1 sequences from *C. auratus* red var

**Fig. 1:**
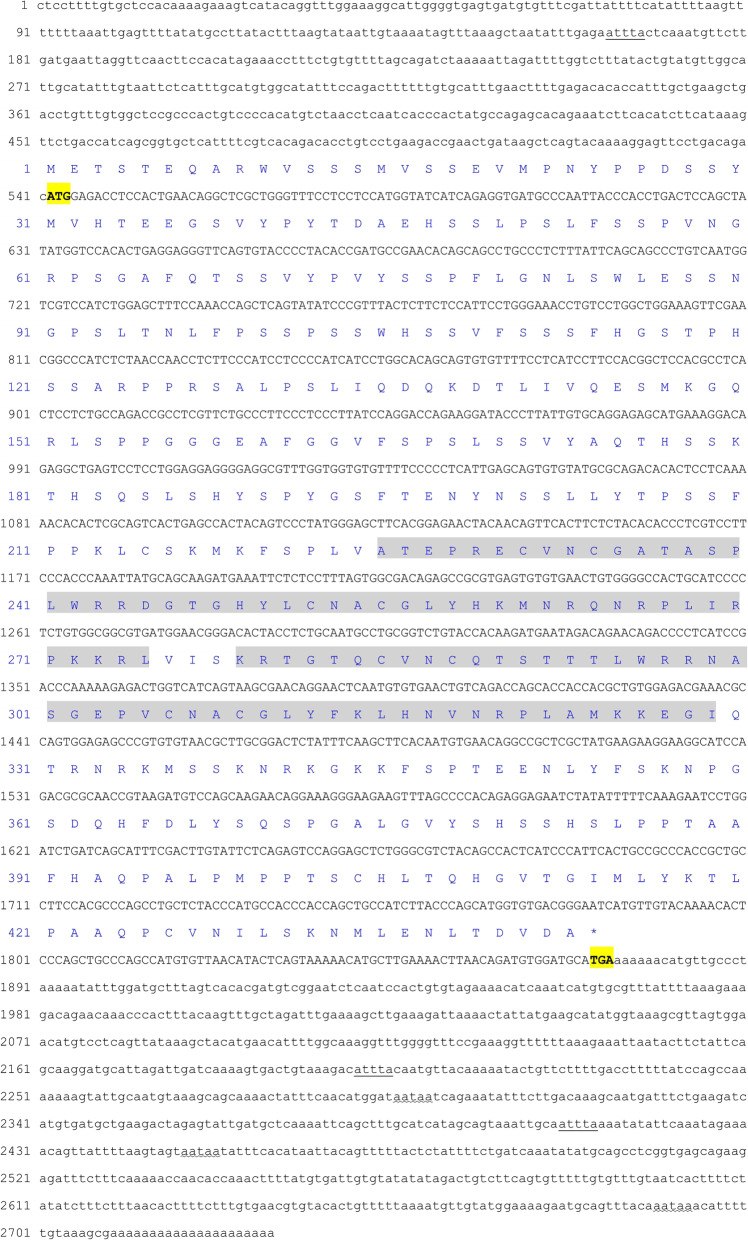
Nucleotide and putative amino acid sequences of *GATA*1and its product. The sequences numbers of nucleotide (lower row) and putative amino acid (upper row) are shown on the left. The translation initiation codon (ATG), stop codons (TGA) are in bold and yellow background. The motif associated mRNA instability (ATTTA) is doubly underscored, and poly-adenylation signal sequence (AATAA) is emphasized by wavy line. The ZnF domains are marked with gray background

The cDNA sequence of *GATA*1 from *C. auratus* red var. (GenBank Accession no. MT322308) is 2730 bp in length, with an ORF of 1332 bp encoding 443 amino acids (aa), 541 bp 5′-UTR, 857 bp 3′-UTR with three poly (A) signal sequences (AATAA), three RNA instability motifs (ATTTA), and a poly (A) tail. Two ZnF domains (aa 225–275, aa 279–329) were predicted in GATA1 protein (Fig. 1).

**Fig. 2:**
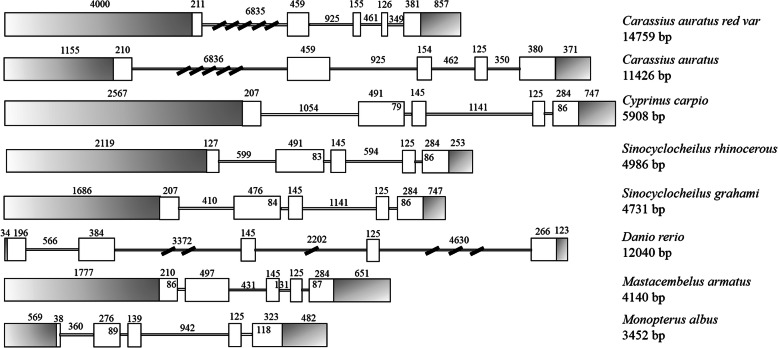
Genomic structure of *GATA*1 genes. The lengths of the elements are shown in base pairs (bp), and the numbers above and below each schematics represent the lengths of exons and introns of corresponding gene, respectively

The genomic sequence of *GATA*1 from *C. auratus* red var. is 14,759 bp in length, which contains 5 exons and 4 introns following the consensus rule of GT/AG (Fig. 2). Comparison of *GATA*1 genomic structures among *Carassius auratus* (Gene ID: 113081347), *Cyprinus carpio* (Gene ID: 109098530), *Sinocyclocheilus rhinocerous* (Gene ID: 107749468), *Sinocyclocheilus grahami* (Gene ID: 107581944), *Danio rerio* (Gene ID: 564960), *Mastacembelus armatus* (Gene ID: 113130813) and *Monopterus albus* (Gene ID: 109968602) demonstrated that the genomic structure of *GATA*1 from *C. auratus* red var. is identical to the *GATA*1 from other teleost fish, all consisting of 5 exons and 4 introns.

### Multiple alignments and phylogenetic analysis

**Fig. 3:**
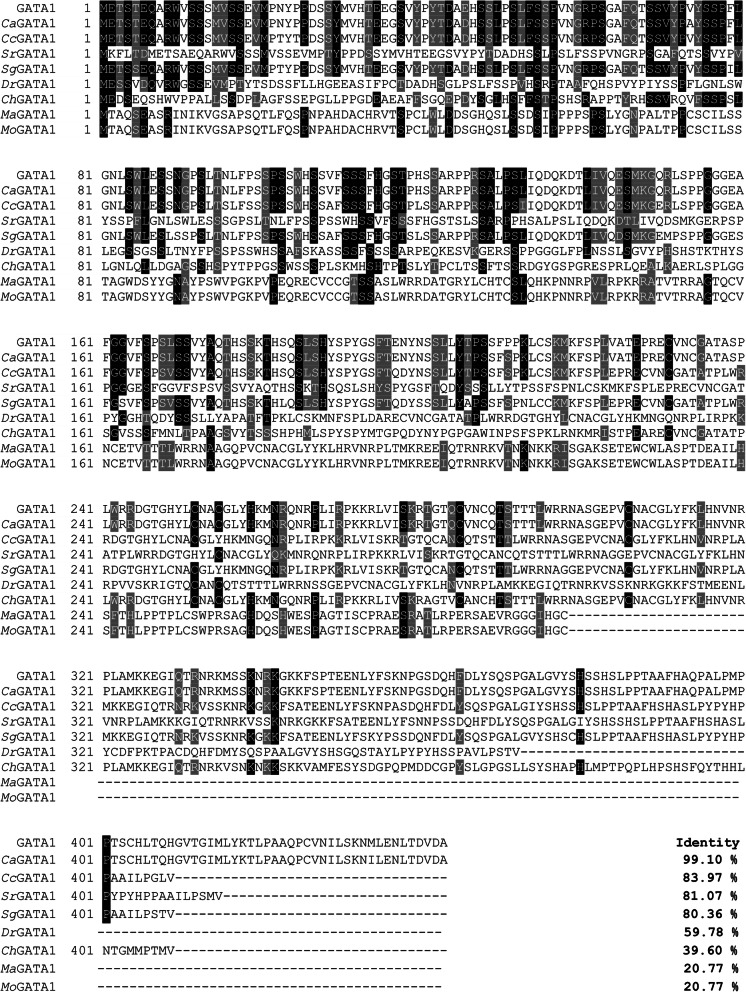
Multiple alignments of GATA1 with GATA1 proteins from various species. The amino acid sequences of GATA1 from typical organisms were aligned using the ClustalW 2.1 program. The black shade represents 100% identity, dark gray represented 80% identity. *Ca*GATA1 stands for GATA1 protein in *Carassius auratus* (Protein ID. XM_026253445.1), *Cc*GATA1 stands for GATA1 protein in *Cyprinus carpio* (Protein ID. XM_019103428.1), *Sr*GATA1 stands for GATA1 protein in *Sinocyclocheilus rhinocerous* (Protein ID. XM_016537268.1), *Sg*GATA1 stands for GATA1 protein in *Sinocyclocheilus grahami* (Protein ID. XM_016271639.1), *Dr*GATA1 stands for GATA1 protein in *Danio rerio* (Protein ID. XP_021334219.1), *Ch*GATA1 stands for GATA1 protein in *Chionodraco hamatus* (Protein ID. KP221299.1), *Ma*GATA1 stands for GATA1 protein in *Mastacembelus armatus* (Protein ID. XP_026189425.1), *Mo*GATA1 stands for GATA1 protein in *Monopterus albus* (Protein ID. XM_020614979.1)

BLASTP analysis (Fig. 3) showed that GATA1 in *C. auratus* red var.shared highest similarity to *Ca*GATA1 (99.10%) and *Cc*GATA1 (83.97%), medium similarities to *Sr*GATA1 (81.07%), *Sg*GATA1 (80.36%), *Dr*GATA1 (59.78%), and *Ch*GATA1 (39.6%), and lowest similarities to *Ma*GATA1 (20.77%) and *Mo*GATA1 (20.77%).

**Fig. 4:**
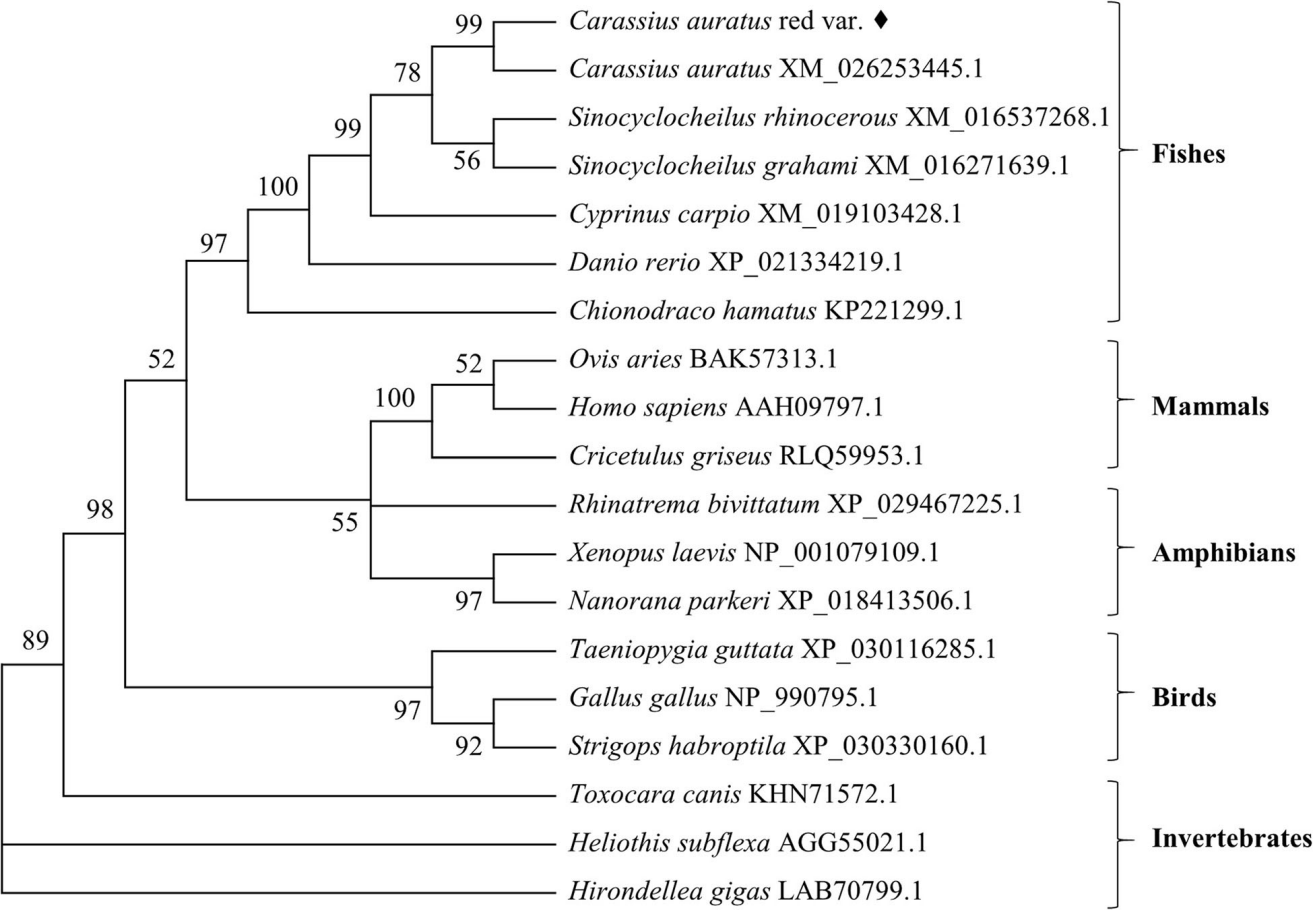
Phylogenetic tree of the GATA1 proteins in different species. A neighbor-joining phylogenetic tree was constructed using MEGA 5.0 software. The bootstrap values of the branches were obtained by testing the tree 1000 times and values were over 50% percent marked. The GenBank accession numbers of GATA1 proteins are given after the species names in the tree

Phylogenetic analysis further supported gene homology among those species (Fig. 4). Homologous amino acid sequences of GATA1 from other teleost fish and non-fish animals were collected from NBCI to construct a phylogenetic tree, which indicated that these homolog proteins could be divided into five groups, representing mammals, birds, amphibians, fishes and invertebrates, respectively. The phylogenetic tree revealed that the GATA1 protein in *C. auratus* red var. is closest to of its ortholog in *C. auratus*, with a high bootstrap value of 99%. All the fish GATA1 proteins clustered together, and diverged from their counterparts in other groups. GATA1 proteins in invertebrates were far separated from those in vertebrates. Thus, the phylogenetic tree reflected a genetic consistency among those species in evolution.

### Distribution of GATA1 in *C. auratus* red var. tissue

**Fig. 5:**
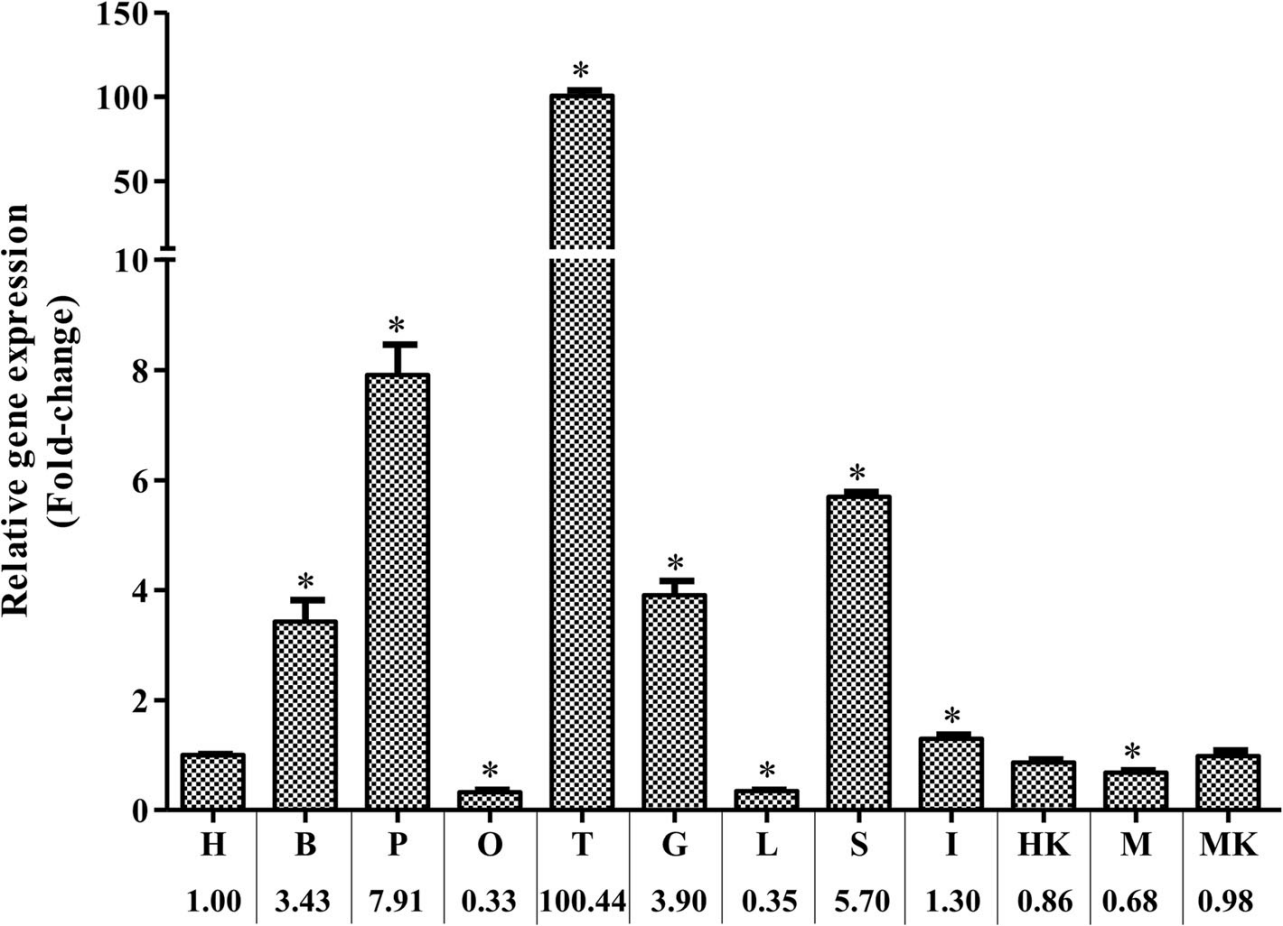
qRT-PCR analysis of the distribution of *GATA*1 in different tissues. Expression of β-actin was used as an internal control for qRT-PCR. The relative expression was the ratio of gene expression in different tissues relative to that in the heart (H). Detailed values are listed at the bottom of the figure. The assay was performed three times, and data were analyzed by the unpaired t-test. *P < 0.05, compared with control

qRT-PCR was performed to analyze the tissue distribution of *GATA*1 mRNA expression. As shown in Fig. 5, *GATA*1 expression was detected in all organs tested, and the values were calibrated against the expression level in heart (H). *GATA*1 had the highest expression level in testis (T) (100.44 folds, *P* < 0.05); intermediate levels in pituitarium (P) (7.91 folds, *P* < 0.05), spleen (S) (5.70 folds, *P* < 0.05), gills (G) (3.90 folds, *P* < 0.05), brain (B) (3.43 folds, *P* < 0.05); and the lowest levels in muscle (M) (0.68 folds), liver (L) (0.35 folds), and ovary (O) (0.33 folds).

### GATA1 expression in different developmental stages after NP treatment

**Fig. 6:**
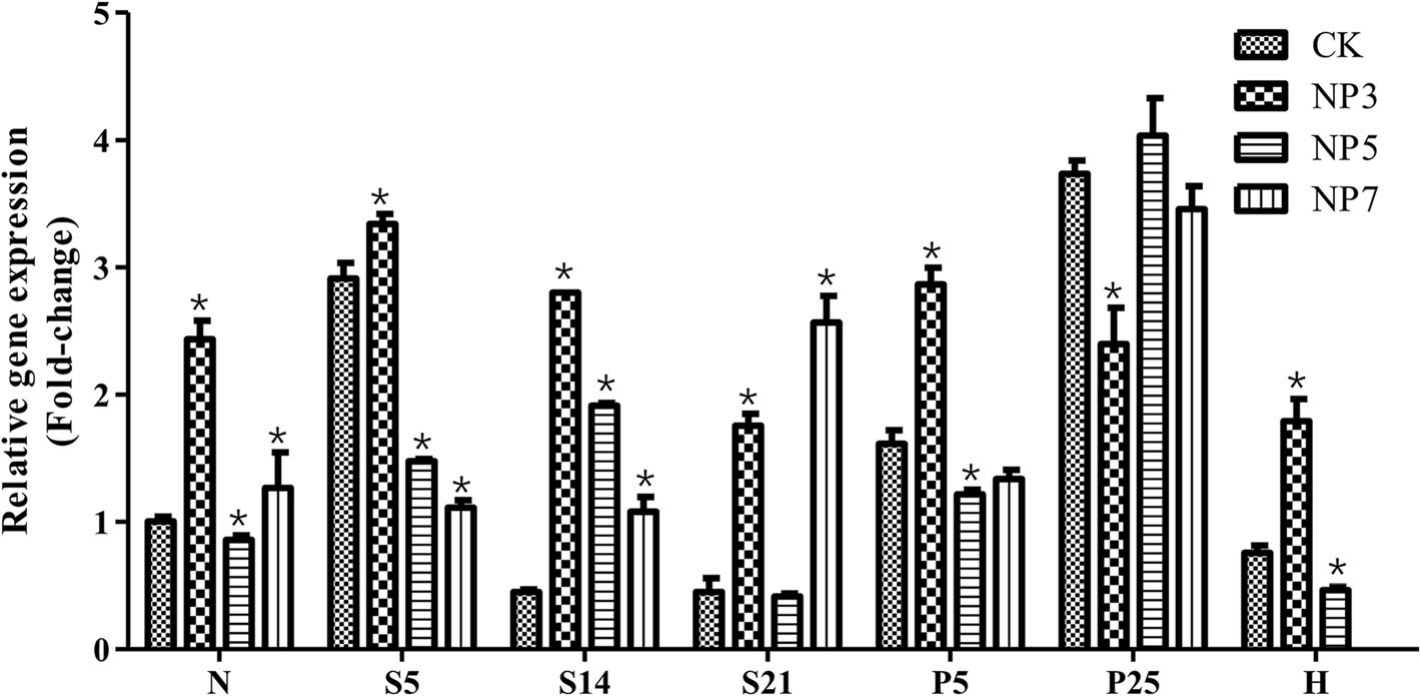
Expression levels of *GATA*1 in the treatment and control groups at various developmental stages. CK: 0 μmol / LNP-treated embryos; NP3: 3 μmol / LNP-treated embryos; NP5: 5 μmol / LNP-treated embryos; NP7: 7 μmol / LNP-treated embryos. *C. auratus* red var. embryos mostly failed to hatch under NP7 treatment, so no H stage samples were collected. The data between different treatment groups at the same developmental period were analyzed by the unpaired t-test. *P < 0.05, compared with control

To determine the effect of NP exposure on *GATA*1 gene expression, the levels of *GATA*1 mRNA in different developmental stages were examined (Fig. 6). During the normal embryonic development, the *GATA*1 gene expression can be detected from the N stage, and the expression level increased at the S5 stage, decreased continuously at S14 and S21 stages, and then increased again at the P5 stage. Afer that, it reached to the highest at the P25 stage, and dropped again after embryo hatching. *GATA*1 expression levels in both the 3 μmol / L and 5 μmol / L NP-treated groups showed the biggest difference from that in the control group at the S14 stage, while the biggest difference in *GATA1* expression between the 7 μmol / L NP-treated and control groups happened at S21 stage. (Fig. 6). On the other hand The biggest difference in GATA1 mRNA levels at the neuroblast stage was found between the control and the 3 μmol / L NP treated groups when compared with other group pairs. When embryos developed to the 5 somite stage, the 7 μmol / L NP-exposure group had greater effect on the expression of *GATA*1 gene than other treated groups with lower NP dosages (Fig. 6). Apparently, NP affected *GATA1* expression during the development of *C. auratus* red var. embryos, and the greatest effect took place in somatic embryos.

### The methylation levels of GATA1 changed significantly in NP -treated groups

**Fig. 7:**
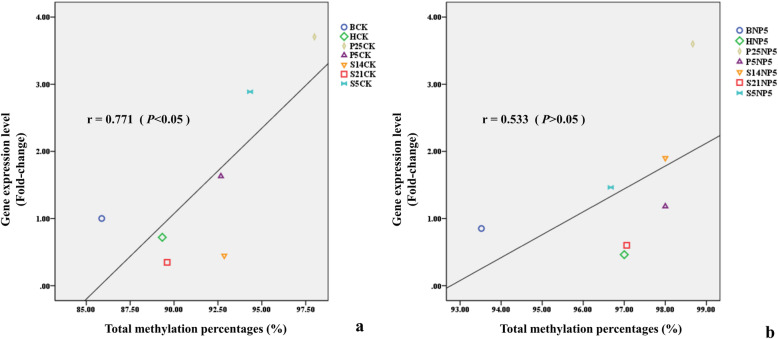
Correlation analysis between *GATA*1 mRNA expression and *GATA*1 gene methylation. a: Scatter plot of correlation between *GATA*1 mRNA expression and methylation degree of 5’UTR region of *GATA*1 gene in control group embryos; b: Scatter plot of correlation between *GATA*1 mRNA expression and methylation degree of *GATA*1 gene 5’UTR region of NP treatment group

Our selected *GATA1*PCR target fragment was 277 bp in size and had 10 CpG sites. Table 1 shows the methylation status of 10 CpG sites in the control and NP5 treatment groups. In the control groups, the methylation rates of *GATA*1 gene at N, S5, S14, S21, P5, P25 and H stages were 85.88, 94.33, 92.86, 89.61, 92.67, 98.00, and 89.33%, respectively. While in the NP-treated groups, the methylation rates of *GATA*1 gene at N, S5, S14, S21, P5, P25 and H stages were 93.52, 96.67, 98.00, 97.06, 98.00, 98.67, and 97.00%, respectively (Fig. 7). Obviously, methylation levels of *GATA*1 gene in the NP-treated embryo groups were mostly higher than that in the control group. We analyzed correlation between *GATA*1 mRNA expression and methylation of *GATA*1 gene in both the control and NP-treated groups, and found *GATA1* expression was significantly positively correlated with its methylation level in the control group (*r* = 0.771, *P* < 0.05), but not in the NP-treated groups (*r* = 0.533, *P* > 0.05).

**Table 1:** Methylation status of the 5’UTR region of the *GATA1* gene

CpG sites	Methylation level of the CK/%	Methylation level of NP5/%
− 3681	100.00	100.00
− 3668	100.00	100.00
− 3657	97.94	100.00
− 3528	86.60	96.08
− 3518	79.38	97.06
− 3490	60.82	69.61
− 3478	89.69	97.06
− 3466	98.79	100.00
− 3434	100.00	100.00
− 3413	100.00	100.00

The CpG sites were located between − 3413 ~ − 3681 upstream to the start codon

CK: 0 μmol / LNP-treated embryos; NP5: 5 μmol / LNP-treated embryos

Degree of methylation = methylation number of the measured CpG sites/total number of the CpG sites measured

## Discussion

In this study, *C. auratust*he full-length *GATA*1 cDNA sequence was obtained from *C. auratus* red var. by homologous cloning and RACE Technology*c. auratus*. *GATA*1 cDNA is 2730 bp in length with a 1332 bp-long ORF encodeing 443 amino acids (aa) a 541 bp-long 5′-UTR and an 857-long bp 3′-UTR. *C. auratus* red var. is a variant of *Carassius auratus*. Alignment analysis revealed that the similarity between the *C. auratus* red var. GATA1 and *C. auratus* GATA1 protein was as high as 99.1%. Also, phylogenetic analysis showed that the GATA1 protein in *C. auratus* red var. was closest to that in *C. auratus*, with bootstrap values reaching 99%. The high similarity between the *C. auratus* red var. and *C. auratus* amino acid sequences is in line with our expectations. In vertebrates, members of the GATA family generally consist of five or six exons and include two conserved type IV zinc finger domains: an amino terminal zinc finger (N) and a carboxyl terminal one (C) [24]. We analyzed the conserved domains in the predicted GATA1 protein with SMART, and found that the *C. auratus* red var. GATA1 contains two ZnF domains (aa 225–275, aa 279–329); The *C. auratus* red var. *GATA*1 gene consists of five exons, which is consistent with its paralogs in other species.

The tissue distribution of *GATA*1 mRNA was analyzed through qRT-PCR. *GATA*1 expression was detected in all tested tissues of *C. auratus* red var. *GATA*1 had the highest expression level in testicle (T); intermediate levels in pituitarium (P), spleen (S), gills (G), brain (B); and lower levels in muscle (M), liver (L), and ovary (O). *GATA*1 is abundantly transcribed in mouse testis and regulates genes involved in the earliest stages of spermatogenesis [25]. Studies have shown that spermatogenesis is induced by GATA-1 expression in Sertoli cells. As atranscription factor, GATA-1 is a developmental stage- and spermatogenic cycle-specific regulator of gene expression in Sertoli cells [26]. In sexually mature *C. auratus* red var. individuals used in this study, the *GATA*1 expression is most enriched in the testes among all tissues and organs. The GATA transcription factor family is essential for pituitary cell differentiation and gonadotropin subunit expression [27]. *GATA*1 inhibits formation of rat cortical neurons, and *GATA1* overexpression ofin the hippocampus can cause depressive behavior in rats [28]. Spleen is an important hematopoietic organ in animal bodies, and the gills are the respiratory fish organs with a large number of capillaries. Accordingly as a key regulator of red blood cell production [18], the *GATA*1 gene is expressed at high levels in P, S, G, and B of *C. auratus* red var. We also found very low *GATA1* expression in L and O of *C. auratus red var*. which was consistent with a report in Nile tilapia (*Oreochromis niloticus*) [29]. *C. auratus*. Thus, our results with *GATA1* expression pattern in various tissues and organs *C. auratus* provided essential cues to understand *GATA*1 functions in *C. auratus* red var. adults.

In *C. auratus* red var. embryos, the *GATA*1 gene starts to be detectable from the neural embryo stage, and stays continuously expressed during embryonic development with relatively stable expression levels, which indicates that *GATA*1 is involved in the entire embryonic development of *C. auratus* red var. *GATA*1is also involved in early embryonic development in other fish. In general, early blood islands emerged in the yolk sac endoderm and splanchnic mesoderm during early embryonic development. *GATA* expression became detectable in zebrafish ectoderm 9 h after fertilization [30]. In *Branchiostoma belcheri*, *GATA*1 expression signal could also be detected at the mesendoderm of gastrula stage [31]. NP affects *GATA*1 expression during the development of *C. auratus* red var. embryos, *C. auratus* with a strongest effect at the somatic stage, and with a most effective NP concentration at 3 μmol / L*C. auratus*. During vertebrate early embryogenesis, the ventral development is directed by the ventral-to-dorsal activity gradient of the bone morphogenetic protein (BMP) signaling [32]. Abnormalties in the BMP signaling pathway may cause strong dorsalization phenotypes in embryos [33]. *GATA*1 gene is a downstream target of the BMP signaling pathway [34], and is shown to exert repressive effects on spine formation in cortical neurons [35]. Under NP stress, the abnormal expression of *GATA*1 gene in *C. auratus* red var. embryos may count for dorsalization after NP treatment. The transcriptional activity of GATA1 is related to the expression level of vitellogenin (*Vg*) [36]. *Vg* expression measurement has been used as a biomarker of exposure to endocrine-disrupting chemicals [37]. Up-regulation of *GATA*1 expression in *C. auratus* red var. embryos under NP exposure may increase *Vg* expression, further proving NP is an environmental endocrine disruptor. Yokomizo et al.’s experiments in mouse embryos provided evidence showing the presence of GATA-1(+) hemangioblastic cells in the extra-embryonic region, demonstrating that the *GATA*1 is involved in definitive hematopoiesis at embryonic stage in close association with endothelial development [38]. *GATA*1 or *GATA*2 is required to initiate blood production in the embryo, so *GATA*1 and *GATA2* double deficient mice exhibit no visible blood cells [39]. In addition, *GATA*1 mutations lead to increased thrombosis in mice [20]. Therefore, the occurrence of thrombosis in *C. auratus* red var. embryos under NP stress may be related to down-regulation of *GATA*1 gene expression.

DNA methylation is a heritable modification that affects gene expression without changing DNA sequences. This modification is crucial to embryonic development. Either abnormally higher or lower methylation levels could be detrimental to the normal growth and embryonic development [40]. Reduced methylation on H3-K4 in Lsd1 mutant fruit flies results in tissue-specific developmental defects [41]. Compared with that in normal embryos, *H*19 gene methylation is severely altered in abnormally developing embryos [42]. In offspring of vitamin-deficient rats, the embryos showed a higher incidence of heart defects, possibly due to the high methylation level of the *GATA*4 gene [43]. In this study, we found that the methylation level of *GATA*1 in the control group was lower than that in the NP-treated *C. auratus* red var. embryos, which demonstrates that NP stress increases methylation level of *GATA*1 in *C. auratus* red var. during embryonic development. *GATA*1 expression is significantly positively correlated with its methylation level in the control group, but not in the NP-treated group. In addition, high levels of *GATA*1 expression during the same developmental period are not necessarily low in methylation level. Since *GATA1* expression levels at different developmental stages in the control group were apparently correlated with *GATA1* methylation levels, we suspected that NP exposure affected *GATA1* expression through changing its methylation status. However, due to the lack of correlation between *GATA1* expression and its methylation in the NP-treated groups, other mechanisms must have been involved. *C. auratus* This is similar to what Okada et al. reported in mice. In 3 T3-L1 preadipocytes, demethylation did not increase *leptin* gene expression, and the diet-induced up-regulation of *leptin*, *Mest/Peg1*, and *sFRP5* gene expression in white adipose tissue (WAT) during the development of obesity in mice is not mediated directly by changes in DNA methylation [44]. In addition, when studying the effect of monomeric and oligomeric flavanols (MOF) consumption on the gene expression profile of leukocytes, Milenkovic et al. found that daily supplementation with 200 mg MOF for 8 weeks modulates the expression of genes associated with cardiovascular disease pathways without major changes of their DNA methylation status [45].

## Conclusions

In this study, the full-length cDNA sequence of *GATA1* gene in *C. auratus* red var. was cloned, and the special and temporal expression patterns of *GATA*1 gene in various tissues/organs and embryonic developmental stages of *C. auratus* red var. were analyzed. Changes in *GATA1* expression during NP-stressed embryonic development was measured, which revealed a role of NP-stress in regulation of *GATA1* expression. It provides important cues for unravel *GATA1* functions in fish development and molecular mechanisms through which NP leads to abnormal development in fish embryos.

## Methods

### Fish and sampling

Two-year-old healthy *C. auratus* red var., weighting about 200 ± 10 g with an average length of 15 ± 3 cm, were obtained from the Engineering Research Center of Polyploid Fish Breeding and Reproduction of the State Education Ministry at Hunan Normal University. All experiments performed were approved by the Animal Care Committee of Hunan Normal University. Before experiments, the fish were acclimatized in an indoor freshwater tank at 25 ± 1 °C and fed twice daily with a commercial diet for 1 week. After no abnormal symptoms were observed, the *C. auratus* red var. were subjected to further study.

Three healthy fish were sacrificed as one group, and samples from the gills (G), liver (L), spleen (S), intestines (I), middle kidney (MK), muscle (M), head kidney (HK), heart (H), brain (B), pituitarium (P), and gonads (testis (T) or ovary (O)) were collected, respectively. All samples were immediately homogenized in TRIzol reagent (Invitrogen, USA) and stored at − 80 °C until RNA extraction. At the same time, fin tissues were isolated and fixed in 95% ethanol. To minimize suffering, 100 mg/L MS-222 (Sigma-Aldrich, St Louis, MO, USA) was used to anaesthetize fish before dissection.

### NP treatment

NP was used for challenge experiments. All the embryos 2 min after fertilization were exposed to NP with the concentrations of 0 μmol/L (blank control, 0.01% ethanol), 3 μmol/L, 5 μmol/L and 7 μmol/L, respectively. Each group was employed for 5 parallel repetitions. Embryo incubation and NP exposure were carried out in 25 cm glass at 25 ± 1 °C.

Intact embryos were collected at 7 stages: neuroblast stage (N), 5 somite stage (S5), 14 somite stage (S14), 21 somite stage (S21), pharyngeal stage-primordium-5 (P5), pharyngeal stage-primordium-25 (P25) and hatching stage (H) after NP exposure. Six groups with 30 embryos in each group were collected at each time points and we used liquid nitrogen to stop embryo development. Samples within each group were homogenized, and aliquots of homogenized tissues were taken for DNA and RNA isolation, respectively.

### RNA extraction and cDNA synthesis

The total RNAs were extracted according to the manufacturer’s instruction for TRIzol reagent. Later, the RNA samples were incubated in RNase-free DNase I (Promega, USA) to eliminate any contaminating genomic DNA. Random primers and a ReverTra Ace kit (Toyobo, Japan) were used for reverse transcription to generate cDNA. Samples that need to be extracted total RNAs include: various tissues of healthy adult fish, embryos of the treatment group and the control group at different developmental stages. SMART™ RACE cDNA Amplification Kit (Takara, Japan) was used to obtain 5′-RACE Ready cDNA and 3′-RACE Ready cDNA.

### Full-length cDNA cloning and analysis

To identify the cDNA sequence of *GATA*1 from *C. auratus* red var., primers GATA1-F1/R1 (Table 2) were designed and synthesized based on the highly conserved regions of known fish *GATA*1 sequences, including *Carassius auratus GATA*1 (*CaGATA*1, Accession no. XM_026253445.1) and *Sinocyclocheilus rhinocerous GATA*1 (*SrGATA*1, Accession no. XM_016537268.1). The 5′ and 3′ untranslated regions (UTRs) were obtained according to the manufacturer’s instruction for SMART™ RACE cDNA Amplification Kit. The full-length cDNA sequences were amplified by PCR using GATA1-F2/R2 primers (Table 2) within the 5′and 3′UTRs, respectively.

**Table 2:** Primers for full-length cDNA cloning and qRT-PCR

Primer name	Sequence (5′ → 3′)	Application
GATA1-F1	GCTCCACAAAAGAAAGTCAT	partial sequence obtaining
GATA1-R1	ACGAGGGTGTGTAGAGAAGT	
GATA1-F2	CCTCAATCACCCACTATGCC	ORF qualifying
GATA1-R2	GTGGATTGAGATTCCGACAT	
GATA1-R-out	GCTCTGGCATAGTGGGTGATTGAGGTTA	5′-Race PCR amplification
GATA1-R-in	ATAATCGAAACACATCACTCACCCCA	
GATA1-F-out	GGCGTCTACAGCCACTCATCCCATTCAC	3′-Race PCR amplification
GATA1-F-in	GGATGCTTTAGTCACACGATGTCGGAAT	
GATA1-qF	CCTTCCCTCCCTTATCCAG	qRT-PCR amplification
GATA1-qR	GGTAGTGTCCCGTTCCATC	

Sequence Manipulation Suite (STS) (http://www.bio-soft.net/sms/) was used to analyse the sequences of *GATA*1 from *C. auratus* red var.. The BLASTP program (https://blast.ncbi.nlm.nih.gov/Blast.cgi) was used to search for GATA1 protein sequence from other species in the NCBI (http:// www.ncbi.nlm.nih.gov/). Multiple sequence alignments were performed by the ClustalX 2.1 program (http://www.ebi.ac.uk/tools/ clustalx2.1). Simple Modular Architecture Research Tool (SMART) (http://smart.embl-heidelberg.de/) was used to predict the protein domain features. A phylogenetic tree was constructed by the neighbor-joining (NJ) algorithm embedded in Mega 5.0 software (http://www.megasoftware.net/ index.html) with a minimum of 1000 bootstraps.

### Genomic sequence cloning

Genomic DNA (gDNA) was extracted from the tail fin using the Universal Genomic DNA Kit (CWBio, China) according to the manufacturer’s instructions. Based on the cDNA sequences of *GATA*1, primers (Table 3) were designed to amplify the genomic sequences gradually. Five overlapping fragments were amplified from gDNA and sequenced.

**Table 3:** Primers for genomic DNA sequences

Primer name	Sequence (5′ → 3′)	Product Length (bp)
GATA1-gDNA-F1	CAATCACCCACTATGCCAGAGC	914 bp
GATA1-gDNA-R1	GCTGAATAAAGAGGGCAGGCTG	
GATA1-gDNA-F2	TGGTCCACACTGAGGAGGGTTC	1238 bp
GATA1-gDNA-R2	GGAAACTGTGTACCAGGGACGG	
GATA1-gDNA-F3	CTGAGCCACTACAGTCCCTATG	1172 bp
GATA1-gDNA-R3	AGGGGTCTGTTCTGTCTATTCA	
GATA1-gDNA-F4	GATGGAACGGGACACTACCTCT	664 bp
GATA1-gDNA-R4	TAGAGTCCGCAAGCATTACACA	
GATA1-gDNA-F5	GGAACTCAATGTGTGAACTGTC	528 bp
GATA1-gDNA-R5	CTGTTCTTGCTGGACATCTTAC	

The 5 ‘unknown sequence of the *GATA*1 gene was obtained from the existing gDNA sequence using the Genome Walking Kit (Takara, Japan) according to the manufacturer’s instructions. The gDNA sequence was confirmed by sequencing the PCR product amplified by primers (Table 4) within the 5′ unknown sequences.

**Table 4:** Primers for 5′unknown sequences

Primer name	Sequence (5′ → 3′)	Application
GATA1-SP1	CAGAGCAAGGCTGTGGAAGTCATTT	5′- Walking PCR amplification
GATA1-SP2	GTCCTGGTTTGGAGGTTGTTTGCC	
GATA1-SP3	GCTTCCACCTTTGATAGAGGCTGA	
GATA1-F3	ATGGCTGTAGTGCTCATTCATCGCT	verification
GATA1-R3	CAAGAGATTCACAACTATGACTGCG	

### Quantification of gene expression

qRT-PCR was carried out in StepOnePlus Real-Time PCR System (ABI, USA) to quantify the mRNA expression of *GATA*1 in different tissues, including intestine (I), liver (L), spleen (S), gills (G), middle kidney (MK), muscle (M), head kidney (HK), heart (H), brain (B), pituitarium (P), testicle (T), and ovary (O). Specific primers (Table 2) were designed for qRT-PCR. The housekeeping gene *β-actin* [46] (Table 5) was utilized as an internal control for cDNA normalization, and the expression level in the heart (H) was used as the baseline (1.0) for qRT-PCR.

**Table 5:** Primers for others

Primer name	Sequence (5′ → 3′)	Application
β-actin -F	GGCCTCCCTGTCTATCTTCC	qRT-PCR
β-actin -R	TTGAGAGGTTTGGGTTGGTC	
GATA1-F4	TTTATTTCGTTGGAGGAGATC	methylation sequence obtaining
GATA1-R4	CGCTATCTAAAATACTTTCCACG	

To determine the effects of NP stress on *GATA1* mRNA expression, the expression levels of *GATA*1 in different developmental stages of *C. auratus* red var. embryos (neuroblast stage (N), 5 somite stage (S5), 14 somite stage (S14), 21 somite stage (S21), pharyngeal stage-primordium-5 (P5), pharyngeal stage-primordium-25 (P25) and hatching stage (H)) treated with different concentrations of NP (0 μmol/L, 3 μmol/L, 5 μmol/L and 7 μmol/L) were analyzed. The housekeeping gene *β-actin* was used as the reference gene, and the *GATA*1expression level in neuroblast stage under 0 μmol/L NP stress was used as the baseline for qRT-PCR (1.0).

Three replicates were performed per sample. Expression levels of corresponding genes were calculated using the 2^–△△*CT*^ method [47]. The *GATA*1 expression levels were measured by one-way analysis of variance, followed by Dunnett’s tests for multiple comparisons using SPSS Statistics 20 software. *P* < 0.05 was considered statistically significant.

### Methylation of the GATA1 from *C. auratus* red var

The genomic DNAs in different developmental stages from the 5 μmol/L NP stress group and control group were extracted, respectively. The DNA was subjected to sulfite modification using the EZ DNA Methylation-Gold™ Kit (Zymo Research, China) according to the manufacturer’s instructions. The software Methyl Primer Express v1.0 was used to design specific primers GATA-F4 / R4 (Table 5) in the 5’UTR region of the *GATA*1 gene. The PCR products were purified by a Gel Extraction Kit (Omega, USA), and the purification products were ligated into pMD19-T vectors (Takara, Japan). The ligation products were then transformed into competent *Escherichia coli* DH5α cells (TransGen, China) and cultured at 37 °C. Positive colonies were selected and sequenced by a Bio-tech company (TIANYI HUIYUAN, China). Fifteen groups of colonies were selected for sequencing at each developmental stage. The sequencing results were sorted and methylation status was analyzed. The degree of methylation was expressed as the percentage of the methylation number of the measured CpG sites to the total number of the methylation sites measured. Correlation analysis was performed on the expression of *GATA*1 mRNA and the degree of methylation in the 5’UTR region of *GATA*1 gene using SPSS Statistics 20 software. The correlation between the two variables was showed by the correlation coefficient (r).

## Supplementary Information

**Additional file 1 Checklist S1.** Completed “The ARRIVE Guidelines Checklist” for reporting animal data in this manuscript.

## Data Availability

Data and materials are available from the authors on reasonable request. The *GATA*1 cDNA sequence is available in the GenBank (Accession number MT322308)

## Abbreviations

NP: Nonylphenol
qRT-PCR: Realtime fluorescence quantitative PCR
G: Gills
L: Liver
S: Spleen
T: Testis
P: Pituitarium
I: Intestines
MK: Middle kidney
M: Muscle
HK: Head kidney
H: Heart
B: Brain
O: Ovary
N: Neuroblast stage
S5: 5 somite stage
S14: 14 somite stage
S21: 21 somite stage
P5: Pharyngeal stage-primordium-5
P25: Pharyngeal stage-primordium-25
H: Hatching stage
STS: Sequence Manipulation Suite
SMART: Simple Modular Architecture Research Tool
NJ: Neighbor-joining
gDNA: Genomic DNA
