# Supplementary material for: Cloning and expression analysis of GATA1 gene in Carassius auratus red var
Source: BMC Genom Data. 2021 Mar 18;22:12. doi: 10.1186/s12863-021-00966-3 (PMC7977614; doi:10.1186/s12863-021-00966-3)

1 the accession number does not currently return any data in GenBank.

Reply: There is no error in the accession number in the article, the scheduled release date is: Feb 16, 2021.


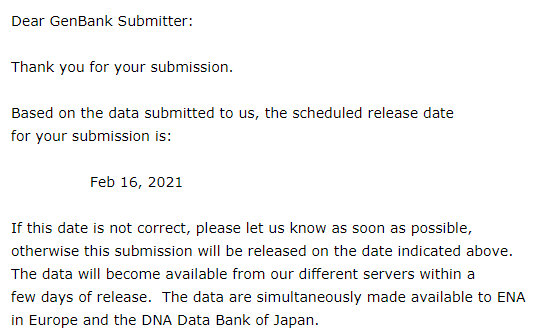


Reply: This data can be found with following link,

https://www.ncbi.nlm.nih.gov/nuccore/MT322308.1/


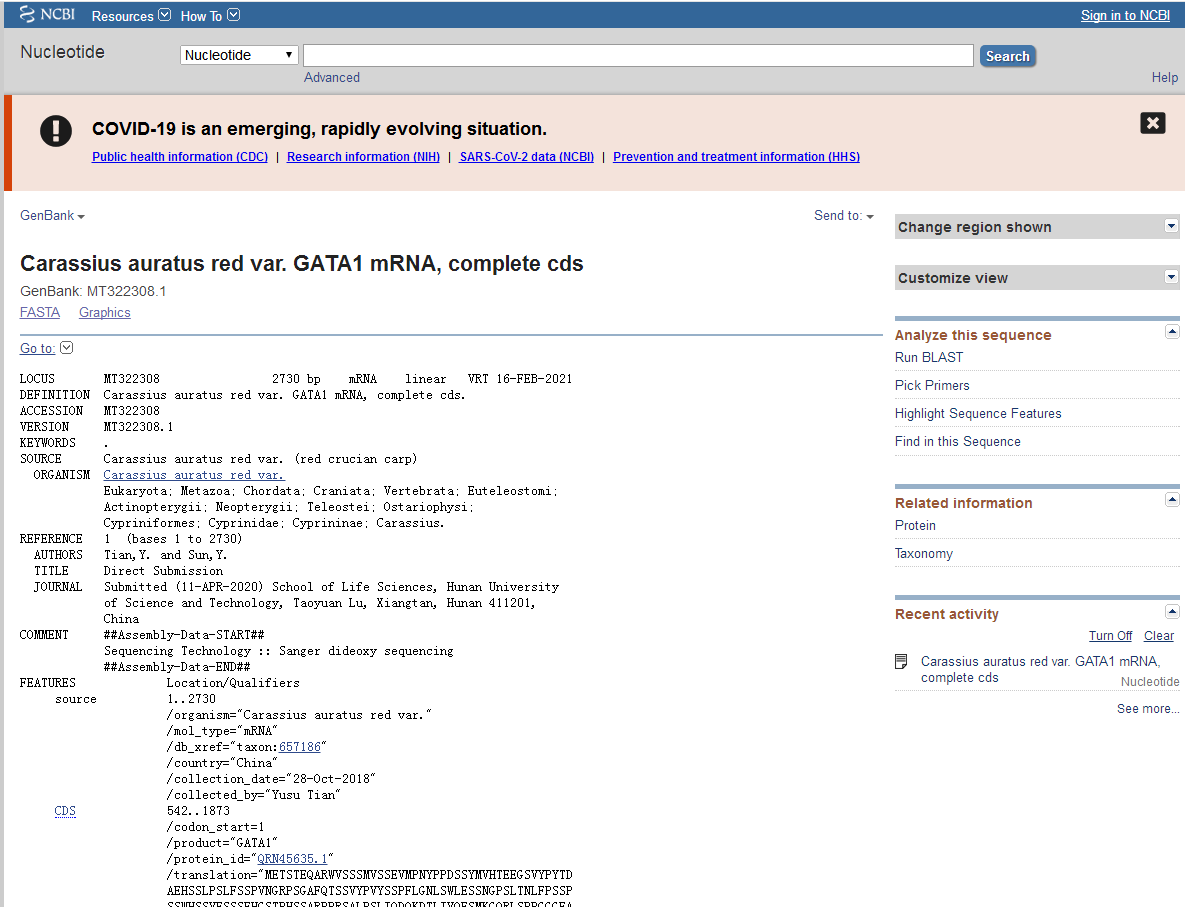


2 The 23rd reference was revised.


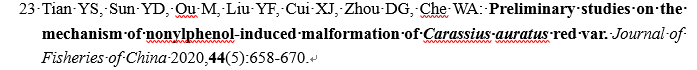


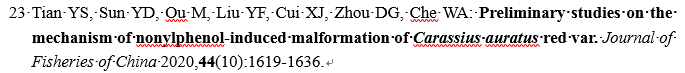


Reply: The previous one was an in-advance online-published version, and the journal has updated it to a formal version, and this is why we also updated it to the lower format as follows,


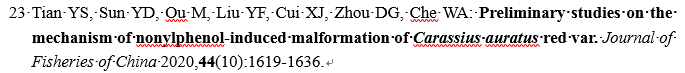

Supplement: Supplementary file 1 — Additional file 1 Checklist S1. Completed “The ARRIVE Guidelines Checklist” for reporting animal data in this manuscript. [file 12863_2021_966_MOESM1_ESM.docx]
